# Exploring the Dynamic Core Microbiome of Plaque Microbiota during Head-and-Neck Radiotherapy Using Pyrosequencing

**DOI:** 10.1371/journal.pone.0056343

**Published:** 2013-02-21

**Authors:** Yue-jian Hu, Zi-yang Shao, Qian Wang, Yun-tao Jiang, Rui Ma, Zi-sheng Tang, Zheng Liu, Jing-ping Liang, Zheng-wei Huang

**Affiliations:** 1 Department of Endodontics, Ninth People’s Hospital, Shanghai Jiao Tong University School of Medicine, Shanghai Key Laboratory of Stomatology, Shanghai, China; 2 Division of Radiation Oncology, Department of Oral and Maxillofacial Surgery, Ninth People’s Hospital, Shanghai Jiao Tong University School of Medicine, Shanghai, China; 3 Department of Endodontics, The Affiliated Stomatology Hospital of Tongji University, Tongji University School of Stomatology, Shanghai, China; Cairo University, Egypt

## Abstract

Radiotherapy is the primary treatment modality used for patients with head-and-neck cancers, but inevitably causes microorganism-related oral complications. This study aims to explore the dynamic core microbiome of oral microbiota in supragingival plaque during the course of head-and-neck radiotherapy. Eight subjects aged 26 to 70 were recruited. Dental plaque samples were collected (over seven sampling time points for each patient) before and during radiotherapy. The V1–V3 hypervariable regions of bacterial 16S rRNA genes were amplified, and the high-throughput pyrosequencing was performed. A total of 140 genera belonging to 13 phyla were found. Four phyla (Actinobacteria, Bacteroidetes, Firmicutes, and Proteobacteria) and 11 genera (*Streptococcus, Actinomyces, Veillonella, Capnocytophaga, Derxia, Neisseria, Rothia, Prevotella, Granulicatella, Luteococcus,* and *Gemella*) were found in all subjects, supporting the concept of a core microbiome. Temporal variation of these major cores in relative abundance were observed, as well as a negative correlation between the number of OTUs and radiation dose. Moreover, an optimized conceptual framework was proposed for defining a dynamic core microbiome in extreme conditions such as radiotherapy. This study presents a theoretical foundation for exploring a core microbiome of communities from time series data, and may help predict community responses to perturbation as caused by exposure to ionizing radiation.

## Introduction

While radiation therapy is a mainstay of treatment for head-and-neck cancers, it is often accompanied by significant side effects. Acute and chronic oral complications that may occur in patients receiving radiotherapy include mucositis, candidiasis, and radiation caries [1], [2]. These side effects probably stem from radiation-induced hyposalivation and consequent shifts in microbial population selected toward a pathogenic flora [3]–[5]. It is widely accepted that the composition of the commensal oral flora is controlled by complex interactions among the oral microorganisms themselves, the host tissues, and the mechanical flushing action and antimicrobial activity of saliva [6]. If one of these factors is greatly altered, changes might be expected to occur in the oral microflora, and disease will manifest itself.

Recent studies of oral microbiota using next generation high-throughput sequencing indicated that there was a core microbiome among oral microbiota phylotypes across communities from different individuals [7]. Taxa found in samples of all time points of all subjects are designated as common [8]. Generally, the term “core microbiome” is defined as the set of members shared among microbial assemblages associated with a particular habitat (e.g. the oral cavity), and is present in all or the vast majority of humans [9]. It has been proposed that the core microbiomes (or common taxa, or shared operational taxonomic units) are likely critical to the ecosystem function of the community within the oral cavity and play a key role in health maintenance. Thus, a better understanding of the composition and variation of these commonly occurring microorganisms is essential to guide the manipulation of communities to achieve desired outcomes and predict community responses to perturbation as caused by exposure to ionizing radiation [10].

Dental plaque harbors a highly diverse resident community of microorganisms. Varying degrees of overlap of plaque microbiota of healthy individuals have been revealed by recent studies of oral microbiome, but little characteristic information of core microbiome in the diseased condition is available so far. Here we hypothesized that radiotherapy of the head and neck induces shifts in the core microbiome of oral microbial communities. Thus, in this study, the high-throughput sequencing technique (pyrosequencing) was used to define the core microbiome of plaque microbiota of irradiated patients compared with the compositional profiles before radiotherapy.

## Materials and Methods

### Ethics Statement

This study was approved by the ethics committee of Shanghai Jiao Tong University and conducted according to the principles expressed in the Declaration of Helsinki. Written informed consent was obtained from all participating patients.

### Enrollment of Subjects

Potential study subjects were identified from a group of patients who were scheduled to receive radiation therapy. Inclusion/exclusion criteria are listed in Table 1. After we had obtained informed consent, the subject population comprised eight individuals who had head-and-neck cancers. Their ages ranged from 26 to 70 years. An oral health examination was performed before radiation therapy. If necessary, carious lesions were restored, endodontic treatment performed, and doubtful teeth extracted. The patients were given oral hygiene instruction, but no special fluoride remedy was added.

**Table 1:** Admission criteria.

Inclusion criteria
a. Presence of caries-free maxillary first molar in the oral cavity and absence of periodontal disease
b. Able to continue current diet and regimen of oral care for duration of the study
c. No anticipated chemotherapy during the course of the study
d. Life expectancy of at least 2 months
e. Eighteen years of age or older
f. Written informed consent
**Exclusion criteria**
a. Untreated cavitated carious lesions or oral abscesses
b. Periodontal pockets ≥4 mm
c. Clinically meaningful halitosis as determined by organoleptic assessment of an experienced clinician
d. Previous diagnosis of Sjögren’s syndrome or any disease characterized by xerostomia
e. Receiving antibiotics during therapy or within 3 months before the study
f. Major salivary glands involved in the surgery region
g. Previous head and neck irradiation
h. Unable to maintain oral hygiene during the study

### Radiation Therapy Protocols

Patients were placed in the supine position on a commercial thermoplastic head-neck mask attached to a carbon-fiber laminate base plate. The transverse images represented 2.5-mm thick slices from 5 cm superior to the skull base to the clavicle heads. The acquired images were transferred directly to a XiO treatment planning system (Computerized Medical Systems, St. Louis, MO, USA). Radiotherapy is concerned with the delivery of the correct radiation dose to the tumour mass. The gray (Gy) is the standard unit of absorbed ionizing-radiation dose expressed in terms of absorbed energy per unit mass of tissue, equivalent to one joule per kilogram (1 J/kg). The primary field was irradiated through lateral parallel-opposed portals with 6-MV photons, 2.0 Gy/30 fractions. Each patient received 10 Gy per week for 6 weeks, with cumulative dose of 60 Gy. The parotid and submandibular glands were directly adjacent to the target volume and could not be spared.

### Microbial Sampling

For each of the eight subjects, before and during radiotherapy, microbial samples were collected at 7-day intervals using the method mentioned in the *Manual of Procedures for Human Microbiome Project* (http://hmpdacc.org/tools_protocols/tools_protocols.php) with minor modifications. Briefly, a sterile Gracey curette was used to collect a pooled supragingival plaque sample from the buccogingival surfaces of the maxillary first molar after the site had been isolated with cotton rolls and dried. The collected plaque sample was released from the curette by agitation in 300 µL of TE buffer (10 mM Tris-Cl [pH 7.5] and 1 mM EDTA). The microbial samples were immediately transported on ice to the laboratory for further DNA extraction and pyrosequencing analysis. All samples were collected at seven time points within 7 weeks. The samples collected at the time point PT (prior to treatment, no dose received) was used as a control group. The following 6-week treatment period included 10 Gy (the first week of radiotherapy), 20 Gy (second week), 30 Gy (third week), 40 Gy (fourth week), 50 Gy (fifth week), 60 Gy (sixth week, the end of radiotherapy).

### DNA Extraction and Pyrosequencing Analysis

The plaque samples were lysed in a Mini-Beadbeater-16 (Biospec Products, Bartlesville, OK, USA) according to the manufacturer’s instructions. The total genomic DNA was obtained from the lysate using a Bacterial Genomic DNA Extraction Kit (QIAGEN, Valencia, USA). All DNA was stored at −20°C before further analysis. PCR amplification of the 16S rDNA hypervariable V1–V3 region [8] was carried out using the forward primer 8F (5′-AGAGTTTGATCCTGGCTCAG-3′) and reverse primer 533R (5′-TTACCGCGGCTGCTGGCAC-3′). Unique barcode sequences were incorporated into the primers so that sequences from different samples can be identified. The PCR program used was as follows: 2 minutes of initial denaturation at 95°C, followed by 20 cycles of denaturation (95°C for 30 seconds), annealing (55°C for 30 seconds) and extension (72°C for 30 seconds) with a final extension of 5 minutes at 72°C. Amplicon pyrosequencing was performed with standard Roche 454 GS-FLX protocols [11]. Raw sequences were processed in a data curation pipeline implemented in the program MOTHUR (version 1.23.1; http://www.mothur.org/). Sequences that were less than 200 bp, contained ambiguous bases or homopolymeric stretches, had a low quality score (<25), or checked as chimeric artifact were discarded after removing the primer sequences and 8-bp barcode. The qualified sequences were submitted to the SILVA database (SILVA 106; http://www.arb-silva.de) for taxonomic analysis. MOTHUR was applied to generate the operational taxonomic units (OTUs) and OTU rarefaction curves. The correlation analyses between the number of OTUs and radiation dose were performed by SAS statistical analysis system (version 8.02; SAS Institute Inc., USA).

## Results

### Overall Sequence Data

**Figure 1:**
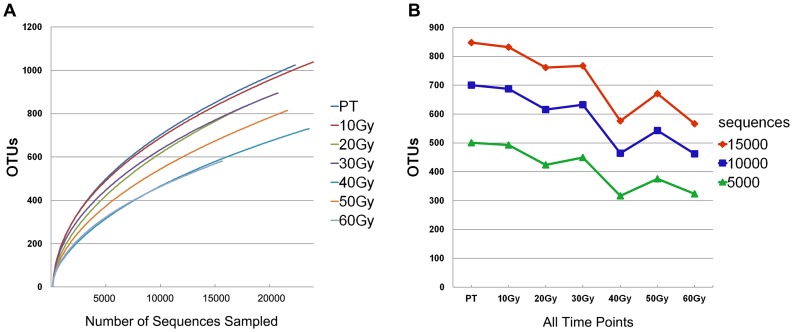
Characterization of all time points rarefaction curves and the correlation analyses between the number of OTUs and radiation dose. (A) Rarefaction curves were used to estimate richness among the seven time points (0.03 dissimilarity level). (B) For a given number of sequences sampled (e.g., 5,000 sequences, 10,000 sequences, or 15,000 sequences), there was a negative correlation between the number of OTUs and dosage (*P*<0.01).

Of 189,305 sequences obtained, 147,232 passed quality control, representing about 78% of the total number of sequences obtained. The average number of sequences per sample at each time point ranged from 1,963 to 3,031 (Table 2). The richness of bacterial communities within supragingival dental plaque at each time point was estimated by rarefaction curves (Figure S1). The curve representing the control group (PT) presented the steepest slope compared with that of the following process of treatment (Figure 1A). Given that the number of sequences obtained from a sample or group had a strong correlation with the number of observed OTUs, comparison of communities from different time point should be made using an equal number of sequences [12]. For a given number of sequences sampled (e.g., 5,000 sequences, 10,000 sequences, or 15,000 sequences; Figure 1B), the control group (PT) had the largest number of OTUs compared with the other time points during radiotherapy (10 Gy–60 Gy). With the radiation dose increasing over time, the distribution of rarefaction curves presented a certain characteristic (Figure 1A). Fewer OTUs were found in the later period (40 Gy, 50 Gy, 60 Gy) than in the early stage (10 Gy, 20 Gy, 30 Gy), and there was a negative correlation between the number of OTUs and dosage (*P*<0.01; Figure 1B). The number of OTUs found across all time points ranged from 165 to 263 by clustering at the 10% dissimilarity level, from 372 to 630 at the 5% dissimilarity level. When a more stringent cut-off was used, more OTUs were formed across all time points, ranging from 580 to 1,038 at the 3% dissimilarity level (Table 2).

**Table 2:** Comparison of number of OTUs at the 3% (0.03), 5% (0.05), and 10% (0.10) dissimilarity level.

Time point	Number of sequences	Average number of sequences per sample (Median, SD)	0.03	0.05	0.10
PT	22580	2823 (2825, 138.8)	1024	617	263
10 Gy	24246	3031 (3051, 148.1)	1038	630	257
20 Gy	18010	2251 (2189, 180.2)	836	524	232
30 Gy	20940	2618 (2655, 144.7)	895	578	247
40 Gy	23865	2983 (2934.5, 183.7)	731	436	182
50 Gy	21887	2736 (2789, 143.3)	815	513	221
60 Gy	15704	1963 (1964, 101.4)	580	372	165

### Composition and Variation of the Bacterial Community

**Figure 2:**
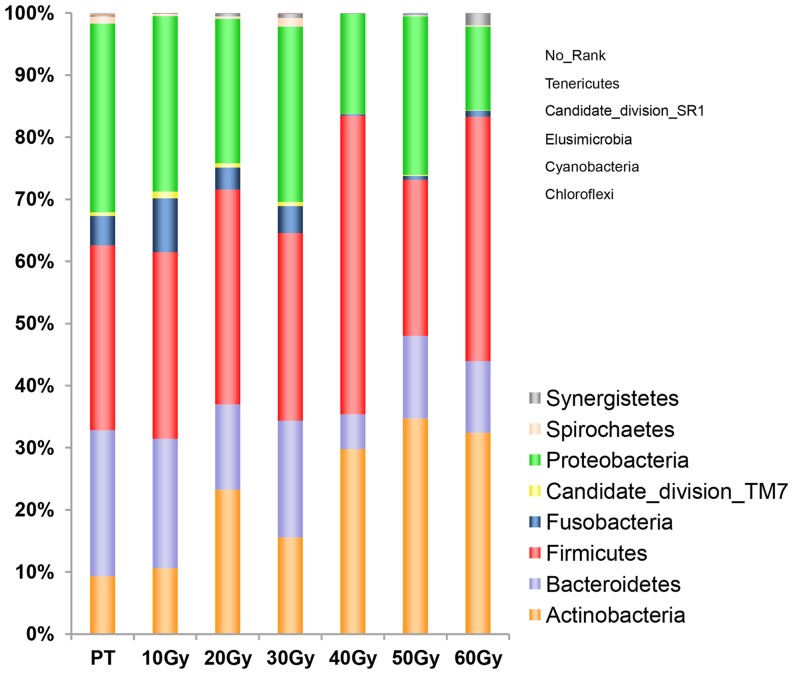
Relative abundance of predominant phyla at seven time points. Members of Actinobacteria, Bacteroidetes, Firmicutes, and Proteobacteria were found at all time points in all subjects and comprised the core microbiome. Phyla having the lowest relative abundance cannot be identified in histogram, and they were listed in small font.

After eliminating unidentified sequences, 13 phyla were found in the oral microbiota at seven time points, which were dominated by eight major phyla (Figure 2). The predominant phyla were Actinobacteria, Bacteroidetes, Firmicutes, Fusobacteria, Proteobacteria, Spirochaetes, Synergistetes, and candidate division TM7. Among them, Synergistetes were identified in six of our subjects, though their richness and relative abundance were very low. The top 4 phyla in the control group (PT) included, in order of prevalence at the time point PT, Proteobacteria (30.38% of the sequences taxonomically assigned at the phylum level), Firmicutes (29.77%), Bacteroidetes (23.52%) and Actinobacteria (9.29%). By contrast, the four most abundant phyla during radiotherapy (10 Gy–60 Gy) were, in order of average prevalence across six time points, Firmicutes (ranging from 25.13% to 48.09% during the course of radiotherapy), Actinobacteria (10.59%–34.7%), Proteobacteria (13.46%–28.24%) and Bacteroidetes (5.6%–20.84%) (Figure 2). The above mentioned four phyla (Actinobacteria, Bacteroidetes, Firmicutes, and Proteobacteria) were found at all time points (before and during treatment) in all subjects and comprised the core microbiome (95% of all sequences). Fusobacteria, Spirochaetes, Synergistetes, and Candidate division TM7 were identified at all time points of some subjects but absent at all time points of other subjects and were designated as subject-specific taxa. The other phyla, such as Chloroflexi, Tenericutes, and Candidate division SR1, which had the lowest relative abundance in sequences, were not found in some time points possibly because their numbers in each sample were below the detection limit of the assay. Other rare phyla like Cyanobacteria were most likely transient “contaminants” of the mouth, gaining access to the oral cavity through food intake or exposure to airborne pollen [8].

**Figure 3:**
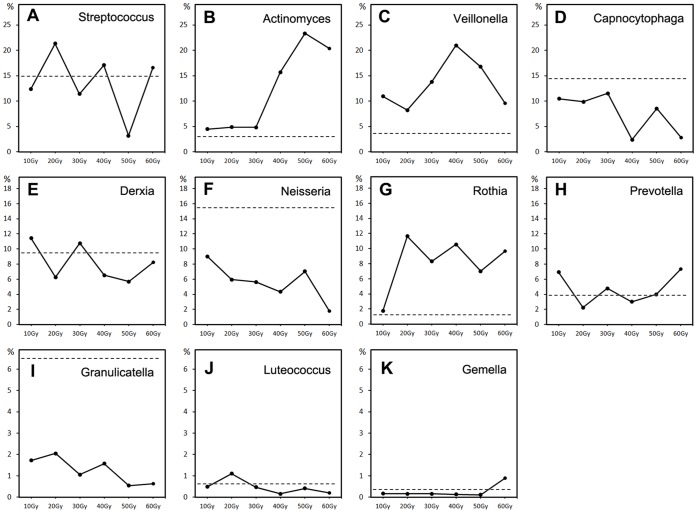
Temporal variation in relative abundance of 11 genera found in all subjects (two common taxa and nine “potential common taxa”). These 11 genera constituted 75.27% of the total sequences. The abscissas and ordinates represent six time points and relative abundance of each genus respectively (different in ordinate scale). The levels of relative abundance of the control group (prior to treatment) are indicated by dotted lines.

At the genus level, sequences from samples of seven time points represented 140 different genera after eliminating the unclassified sequences. The top 10 taxa that constituted roughly 80% of the sequences in relative abundance belonged to *Streptococcus, Veillonella, Actinomyces, Capnocytophaga, Derxia, Neisseria, Rothia, Prevotella, Lactobacillus,* and *Leptotrichia*. Among these, six genera (*Streptococcus, Actinomyces, Capnocytophaga, Neisseria, Granulicatella,* and *Gemella*) were found in the control group (PT) in all subjects and were designated as common at the time point PT. By contrast, only two genera (*Streptococcus* and *Actinomyces*) were present across the time points from 10 Gy to 60 Gy in all subjects. These were designated as “common taxa” during radiotherapy, but were fewer than that in the control group or in previous reports on oral microbial communities of healthy individuals [7], [8]. The relative abundance of *Streptococcus* fluctuated between 21.33% (20 Gy) and 3.2% (50 Gy), and that of *Actinomyces* remained stable (about 4.48% to 4.85%) in the early stage of the time points (10 Gy, 20 Gy, 30 Gy), but rose to 23.32% at the 50 Gy time point. In addition to the two genera mentioned above, several genera including *Veillonella, Capnocytophaga, Derxia, Neisseria, Rothia, Prevotella, Granulicatella, Luteococcus,* and *Gemella* could be identified in all subjects during radiotherapy (10 Gy–60 Gy) but were absent at some time points of each subject. These nine genera could be recognized as “potential common taxa” in this study (discussed below). The most abundant genus at the time point PT was *Neisseria* (15.54%). However, at the time points 10 Gy and 20 Gy, the most abundant genus was *Streptococcus* (12.34% and 21.33%, respectively). At the time points 30 Gy and 40 Gy, *Veillonella* predominated (13.77% and 20.98%, respectively), even though this genus was not found in some samples. At the time points 50 Gy and 60 Gy, *Actinomyces* ranked first in relative abundance (23.32% and 20.38%, respectively; Figure 3). The major genera varied significantly in relative abundance across different time points. About 2.7% (4,038) of all sequences could not be identified at the genus level and were classified at the higher taxonomic levels (family, order, class, and phylum).

At the species level, defined OTUs on the basis of 3% dissimilarity, the number of phylotypes at seven time points ranged from 580 to 1,038, while a more conservative approach–5% dissimilarity level among OTUs–yielded 372 to 630 phylotypes. Individual species or strains were not described because of the relatively short reads and the low taxonomic resolution below the genus level [13], [14]. The rarefaction curves were generated at the 3% dissimilarity level in this study (Figure 1). Generally, the cut-off of 3% dissimilarity was used in species level analyses, which facilitates a direct comparison with other studies [7], [13], [15].

## Discussion

Radiation therapy is the most widely used approach to treat head-and-neck cancers. This technique involves three coplanar isocentric fields designed to adequately cover the target volumes, with the major salivary glands being inevitably exposed to the radiation field [16], [17]. The high radiosensitivity of these glands causes the salivary secretion rate to continuously decrease and the microflora composition of the saliva to change. Saliva is important for a variety of functions in the oral cavity. It contains buffering systems that neutralize the acids formed during bacterial carbohydrate metabolism. Salivary components such as lysozyme, histatins and lactoferrin provide an antimicrobial function crucial to prevent progression of oral diseases [18]. As a result of radiation-induced hyposalivation, an imbalance in the oral microbial ecosystem is inevitable. In addition, the microbial changes may result from a direct or indirect effect of ionizing radiation [19], [20], lifestyle, dietary habit, medical history, or other factors that vary between individuals. In order to eliminate adverse effect brought by inter-individual variations, a self-comparative analysis was proposed to reduce selection biases and achieve more reliable results. Thus, in this study, the samples collected before treatment was used as a control group and compared with the following treatment procedures [14].

One fundamental question raised by sponsored human microbiome projects (HMPs) is whether there is an identifiable “core microbiome” of shared organisms, genes or functional capabilities found in a given body habitat (e.g., the oral cavity) of all or the vast majority of humans [21]. Several models of “core microbiome” have been presented in the past few years [10], [22], but the most typical approach used to identify a core was to report the number of phylotypes (or OTUs) found across samples from a similar habitat and find the overlap of different communities based on a presence/absence data set. For instance, if one OTU was present in all or the vast majority of subjects, this OTU should be considered as a member of the “core” [9], whether it had high or low abundance. The most stringent definition of a core microbiome would require its presence in all subjects (100%) sampled [23]. Zaura et al. obtained the first insight into the oral core microbiome and found the overlap of oral microbial communities from a small population of 3 individuals [7]. Huse et al. explored the core microbiome with a larger sample size [23], but they defined “core” as those shared amongst 95% or more of the subjects and didn’t incorporate time series data (samples obtained at different time points). A recent study presented a more systematic conceptual framework for identifying core microbiomes [10] in which the core microbiome from time series data was comprised of OTUs that were consistently observed across all time points and shared by all subjects (OTU e in Table 3). However, this framework is only suitable for the analyses of communities in the stable or healthy status with little perturbation. Some potential cores may be overlooked under extreme conditions such as radiotherapy or other chemical and physical irritants. A typical example in this study was the assemblage of above mentioned nine genera which could be identified in all subjects but not at all time points during treatment. Actually, the consistent presence of these genera over time was due to computational analysis of pooled data. These genera, defined as core microbiomes in previous studies in healthy individuals [7], [8], were probably potential common taxa and their presences and absences at some time points might be due to radiation-induced changes in the oral ecosystem. Thus, we recommend that taxa detected in all subjects but absent at some time points under extreme conditions (OTU f-i in Table 3) should be considered as a dynamic core microbiome when dealing with the data of time series (suppose that methodological errors are not involved). Briefly, we define the dynamic “core” during radiotherapy as those that are present in all subjects but not necessarily persistent over time (Table S1). Moreover, the lowest depth of sampling in the present study was about 1,963 sequences/sample (60 Gy) which was possibly insufficient to reveal common taxa when present at extremely low abundance. Therefore, our findings on core microbiomes should be considered conservative. It was also possible that sampling of the buccogingival surface of the maxillary first molar as representative of the dental plaque microbiome could not reflect the entire oral microecosystem, because the microbiota might vary depending on the intraoral sites from which the plaque samples were obtained. For example, a higher diversity was likely to be found in the samples taken at the approximal surfaces and the lingual surfaces of the front teeth [7], which could lead to a more complex core microbiome. More efforts are needed to achieve a better understanding of the core microbiome of oral microbial communities.

**Table 3:** The conceptual framework of a dynamic core microbiome in extreme conditions.

	Subject A			Subject B				
	time 1	time 2	time 3	time 1	time 2	time 3		
OTU a	•	•	•				**subject-specific taxa**	
OTU b				•	•	•		
OTU c	•	•					**potential subject-specific taxa**	
OTU d				•		•		
OTU e	•	•	•	•	•	•	**common taxa**	**core microbiome**
OTU f	•	•	•		•	•	**potential common taxa**	
OTU g	•	•		•	•			
OTU h		•	•			•		
OTU i	•			•				

OTU a-i represent the units of interest defined at a given taxonomic level. Subject A and B represent time series communities to be compared using the OTU table. The presences of OTUs are represented by the symbols “•”. The OTUs present at all time points of all subjects are designated as common taxa (OTU e). Those present in all subjects but not at all time points are designated as potential common taxa (OTU f-i). Both taxa (common taxa and potential common taxa) can be recognized as core microbiomes from time series data.

The major challenge facing the various HMPs is how to best relate community composition and microbial changes to physiological impacts. The data in our study showed that the above-mentioned 11 genera (*Streptococcus, Actinomyces, Veillonella, Capnocytophaga, Derxia, Neisseria, Rothia, Prevotella, Granulicatella, Luteococcus* and *Gemella*), which were designated as a core microbiome in this study, varied in relative abundance during the course of radiotherapy (illustrated in detail in Figure 3). *Granulicatella*, for example, is very fastidious and difficult to cultivate. Our study indicates that this genus was dramatically reduced in dental plaque following radiation. It is therefore tempting to speculate that this genus is unlikely to contribute to the pathogenesis of post-radiation diseases such as caries. Meanwhile, a notable fluctuation (e.g., *Streptococcus*) was observed during radiotherapy. *Streptococcus* was one of the predominant genera in oral cavity and consisted of a large numbers of cariogenic and non-cariogenic species, including *S. sobrinus*, *S. mutans*, *S. oralis*, *S. mitis*, and *S. pneumonia*, etc. However, the current technology is generally much more effective in the identification of higher level taxonomic assignments such as phyla, classes, orders, families, and genera [14]. Thus it was difficult to relate the general fluctuation of *Streptococcus* to dental caries. Additional efforts are needed to identify actual species or strains. Furthermore, a change in number of OTUs during radiotherapy was also observed. The negative correlation between the number of OTUs and dosage indicated that there might be a general decrease in number of species as the radiation dose was increased. It is known that oral microbial communities can remain relatively stable over time in healthy individuals [8], [24]. Our results show that dramatic physical and chemical fluctuations (as caused by administration of ionizing radiation) can impose significant changes in the composition of the oral microbiome.

### Conclusion

In summary, the core microbiomes of plaque microbiota in patients receiving head-and-neck radiotherapy were explored by high-throughput pyrosequencing. Among all 13 phyla and 140 genera, 4 phyla and 11 genera were found during radiotherapy in all subjects, supporting the concept of a core microbiome. A negative correlation between the number of OTUs and radiation dose was also observed. Moreover, we proposed an optimized conceptual framework for defining a dynamic core microbiome in extreme conditions such as radiotherapy. This study provides researchers with valuable information about the profiles of oral microbial communities during radiotherapy, and presents a theoretical foundation for exploring a core microbiome of communities from time series data.

## Supporting Information

Figure S1 **Rarefaction curves of seven time points at the 0.03 (3%), 0.05 (5%), and 0.10 (10%) dissimilarity level.**
(PDF)

Table S1 **Numbers of sequences of the core microbiome (11 genera).**
(DOC)
