# Supplementary material for: Exploring the Dynamic Core Microbiome of Plaque Microbiota during Head-and-Neck Radiotherapy Using Pyrosequencing
Source: PLoS One. 2013 Feb 21;8(2):e56343. doi: 10.1371/journal.pone.0056343 (PMC3578878; doi:10.1371/journal.pone.0056343)
Supplement: Figure S1 — Rarefaction curves of seven time points at the 0.03 (3%), 0.05 (5%), and 0.10 (10%) dissimilarity level. (PDF) [file pone.0056343.s001.pdf]

PT Rarefaction Curve

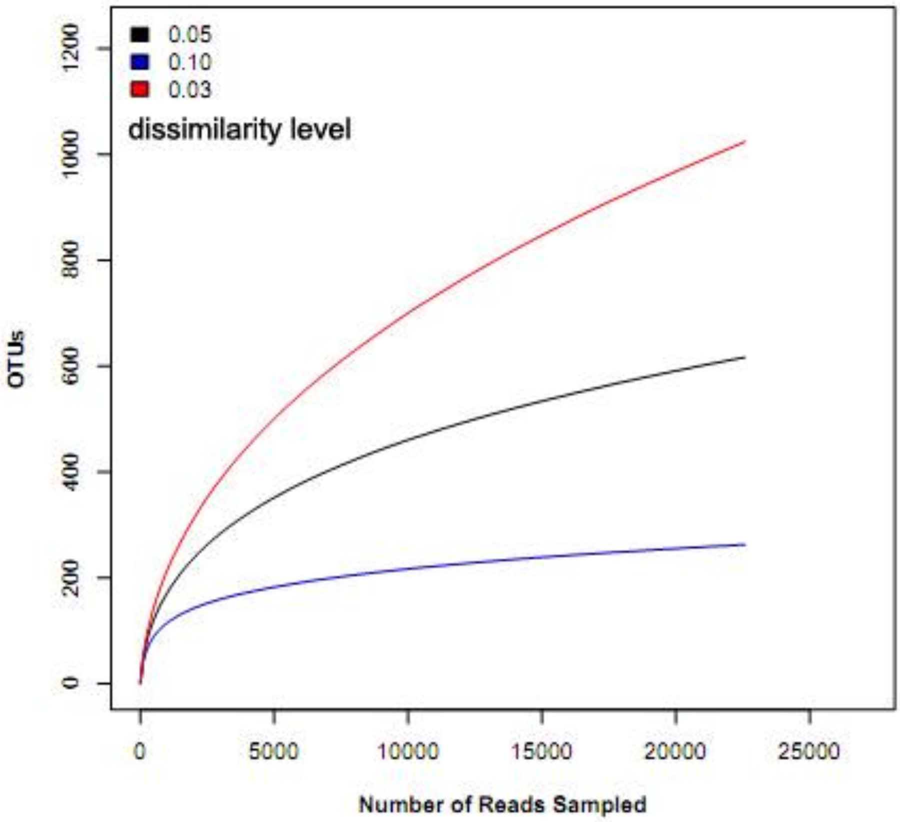

10Gy Rarefaction Curve

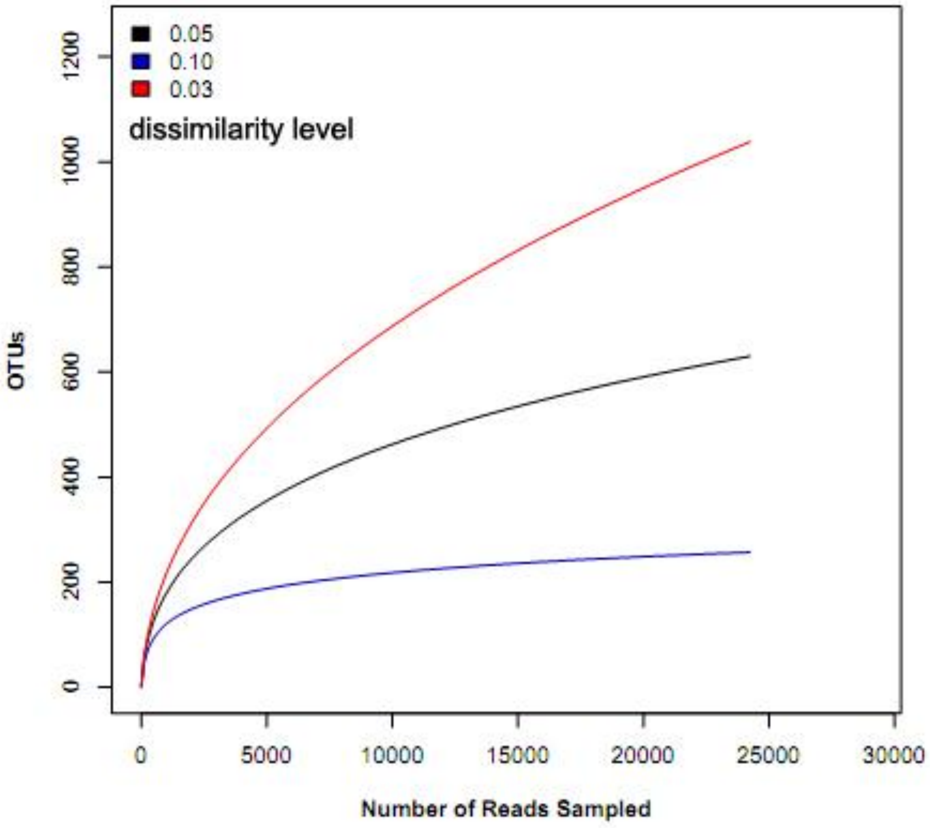

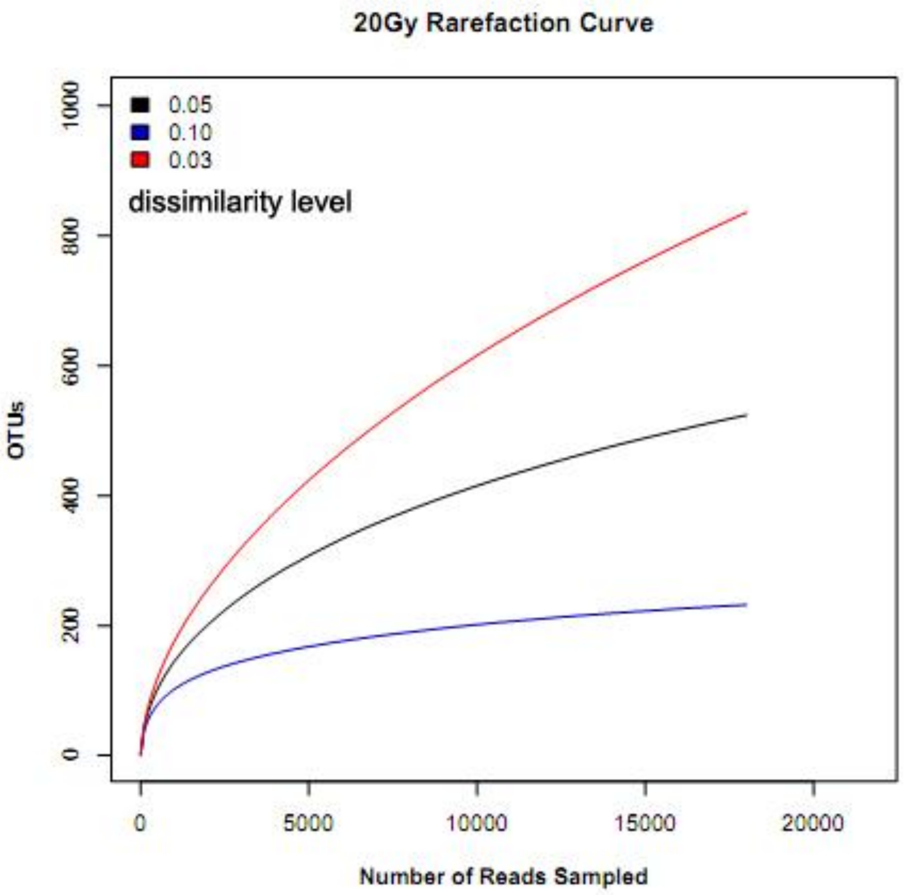
20Gy Rarefaction Curve
0.05
0.10
0.03
dissimilarity level
OTUs
0
200
400
600
800
1000
0
5000
10000
15000
20000
Number of Reads Sampled

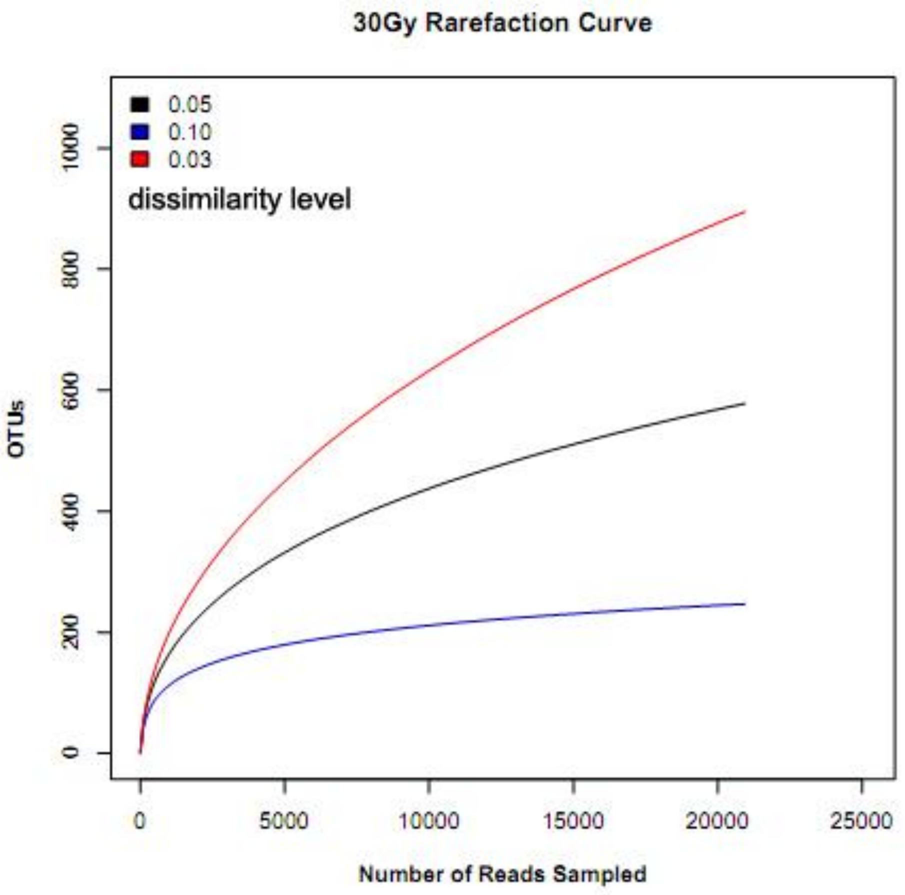
30Gy Rarefaction Curve
0.05
0.10
0.03
dissimilarity level
OTUs
0
200
400
600
800
1000
0
5000
10000
15000
20000
25000
Number of Reads Sampled

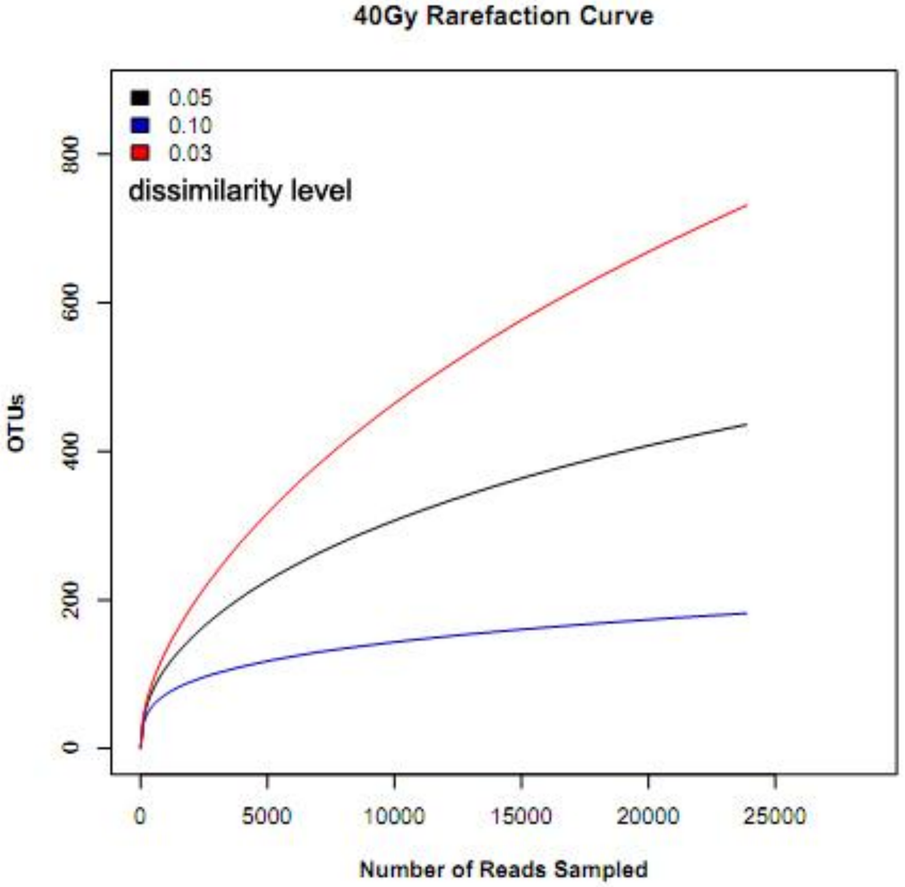
40Gy Rarefaction Curve
0.05
0.10
0.03
dissimilarity level
OTUs
0
200
400
600
800
0
5000
10000
15000
20000
25000
Number of Reads Sampled

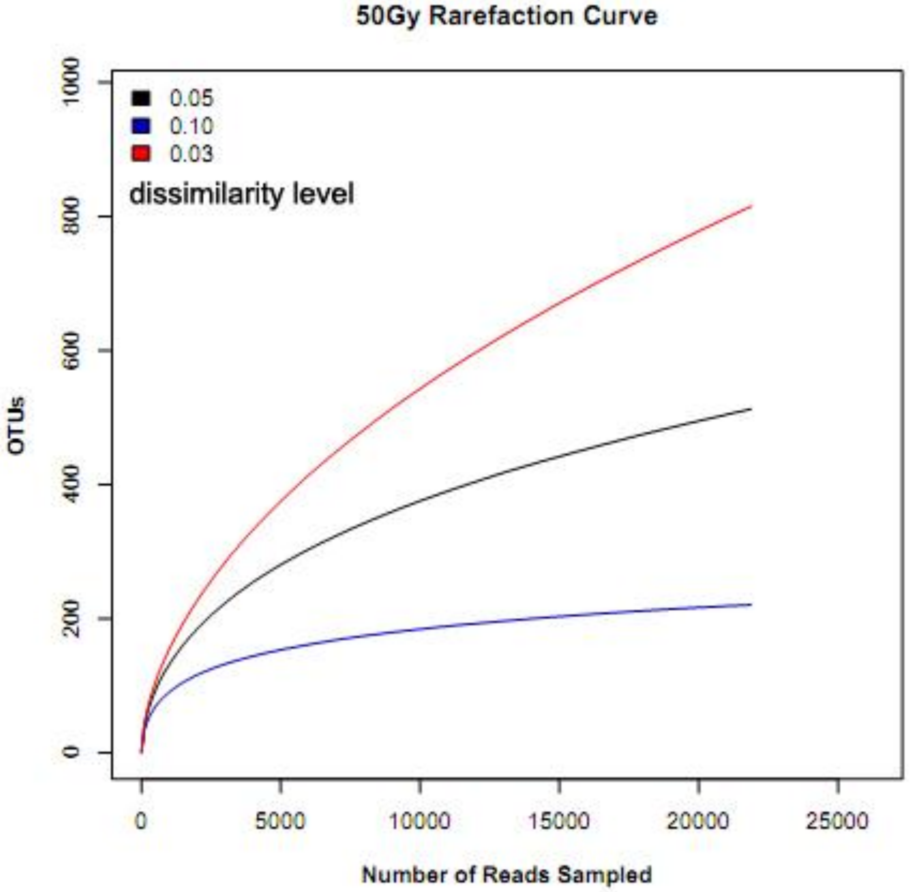
50Gy Rarefaction Curve
0.05
0.10
0.03
dissimilarity level
OTUs
0
200
400
600
800
1000
0
5000
10000
15000
20000
25000
Number of Reads Sampled

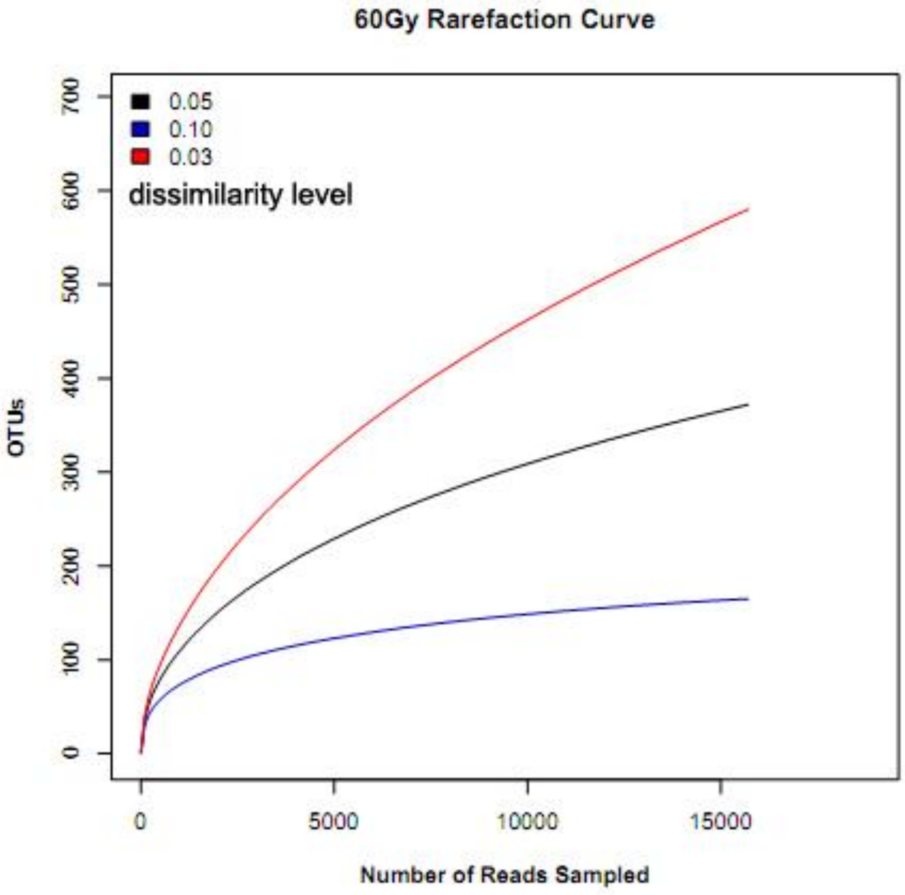
60Gy Rarefaction Curve
0.05
0.10
0.03
dissimilarity level
OTUs
0
100
200
300
400
500
600
700
0
5000
10000
15000
Number of Reads Sampled
