# Supplementary material for: Exploring the Dynamic Core Microbiome of Plaque Microbiota during Head-and-Neck Radiotherapy Using Pyrosequencing
Source: PLoS One. 2013 Feb 21;8(2):e56343. doi: 10.1371/journal.pone.0056343 (PMC3578878; doi:10.1371/journal.pone.0056343)
Supplement: Table S1 — Numbers of sequences of the core microbiome (11 genera). (DOC) [file pone.0056343.s002.doc]

**Supplementary Table S1**

**Numbers of sequences of the core microbiome (11 genera)**

*Streptococcus* and *Actinomyces* were present at all time points of all subjects and were designated as “common taxa”. The rest were present in all subjects but not at all time points, and were recognized as "potential common taxa". Both "common taxa" and "potential common taxa" could be designated as dynamic core microbiome during radiotherapy.

|  | **Subject A** | | | | | | |
| --- | --- | --- | --- | --- | --- | --- | --- |
|  | **PT** | **10Gy** | **20Gy** | **30Gy** | **40Gy** | **50Gy** | **60Gy** |
| Streptococcus | 816 | 539 | 384 | 46 | 99 | 47 | 228 |
| Actinomyces | 8 | 138 | 88 | 291 | 1625 | 767 | 280 |
| Veillonella | 137 | 929 | 148 | 836 | 429 | 179 | 131 |
| Capnocytophaga | 495 | 309 | 178 | 227 | 37 | 15 | 38 |
| Derxia | 620 | 492 | 113 | 225 | 141 | 0 | 113 |
| Neisseria | 347 | 77 | 107 | 32 | 167 | 1 | 25 |
| Rothia | 1 | 0 | 210 | 2 | 40 | 24 | 133 |
| Prevotella | 109 | 179 | 41 | 135 | 506 | 0 | 101 |
| Granulicatella | 182 | 82 | 37 | 19 | 6 | 3 | 9 |
| Luteococcus | 0 | 0 | 20 | 0 | 1 | 1 | 3 |
| Gemella | 4 | 1 | 3 | 8 | 2 | 7 | 12 |
|  |  |  |  |  |  |  |  |
|  | **Subject B** | | | | | | |
|  | **PT** | **10Gy** | **20Gy** | **30Gy** | **40Gy** | **50Gy** | **60Gy** |
| Streptococcus | 750 | 374 | 1317 | 1038 | 1429 | 70 | 625 |
| Actinomyces | 8 | 136 | 19 | 20 | 130 | 511 | 76 |
| Veillonella | 0 | 332 | 57 | 367 | 300 | 367 | 197 |
| Capnocytophaga | 430 | 318 | 0 | 2 | 1 | 187 | 0 |
| Derxia | 2 | 347 | 3 | 0 | 0 | 124 | 0 |
| Neisseria | 585 | 273 | 0 | 2 | 0 | 154 | 0 |
| Rothia | 9 | 54 | 673 | 338 | 82 | 153 | 28 |
| Prevotella | 0 | 210 | 1 | 0 | 3 | 87 | 1 |
| Granulicatella | 956 | 52 | 54 | 26 | 18 | 12 | 10 |
| Luteococcus | 11 | 15 | 0 | 0 | 0 | 9 | 0 |
| Gemella | 5 | 5 | 0 | 1 | 2 | 3 | 0 |
|  |  |  |  |  |  |  |  |
|  | **Subject C** | | | | | | |
|  | **PT** | **10Gy** | **20Gy** | **30Gy** | **40Gy** | **50Gy** | **60Gy** |
| Streptococcus | 355 | 707 | 488 | 243 | 260 | 73 | 25 |
| Actinomyces | 19 | 21 | 76 | 90 | 158 | 311 | 46 |
| Veillonella | 130 | 116 | 748 | 854 | 1802 | 1460 | 384 |
| Capnocytophaga | 484 | 328 | 93 | 68 | 20 | 3 | 7 |
| Derxia | 0 | 1 | 0 | 0 | 0 | 0 | 0 |
| Neisseria | 347 | 14 | 1 | 0 | 2 | 0 | 1 |
| Rothia | 0 | 0 | 254 | 511 | 1022 | 428 | 506 |
| Prevotella | 492 | 294 | 207 | 527 | 5 | 101 | 539 |
| Granulicatella | 69 | 163 | 107 | 17 | 6 | 0 | 2 |
| Luteococcus | 34 | 14 | 16 | 24 | 0 | 2 | 3 |
| Gemella | 21 | 2 | 6 | 0 | 0 | 0 | 0 |
|  |  |  |  |  |  |  |  |
|  | **Subject D** | | | | | | |
|  | **PT** | **10Gy** | **20Gy** | **30Gy** | **40Gy** | **50Gy** | **60Gy** |
| Streptococcus | 232 | 240 | 480 | 358 | 561 | 177 | 326 |
| Actinomyces | 140 | 122 | 110 | 152 | 515 | 137 | 400 |
| Veillonella | 283 | 992 | 185 | 432 | 688 | 929 | 188 |
| Capnocytophaga | 246 | 5 | 223 | 362 | 78 | 182 | 55 |
| Derxia | 12 | 0 | 141 | 338 | 214 | 0 | 161 |
| Neisseria | 536 | 0 | 134 | 177 | 142 | 42 | 35 |
| Rothia | 4 | 0 | 262 | 261 | 347 | 5 | 190 |
| Prevotella | 163 | 946 | 51 | 150 | 98 | 516 | 144 |
| Granulicatella | 25 | 26 | 46 | 33 | 52 | 52 | 13 |
| Luteococcus | 9 | 2 | 25 | 15 | 5 | 0 | 4 |
| Gemella | 4 | 0 | 4 | 5 | 4 | 10 | 18 |
|  |  |  |  |  |  |  |  |
|  | **Subject E** | | | | | | |
|  | **PT** | **10Gy** | **20Gy** | **30Gy** | **40Gy** | **50Gy** | **60Gy** |
| Streptococcus | 216 | 171 | 171 | 53 | 863 | 105 | 359 |
| Actinomyces | 169 | 49 | 21 | 268 | 779 | 766 | 440 |
| Veillonella | 10 | 88 | 93 | 66 | 94 | 550 | 206 |
| Capnocytophaga | 343 | 575 | 823 | 851 | 18 | 280 | 60 |
| Derxia | 1 | 3 | 0 | 0 | 0 | 187 | 178 |
| Neisseria | 261 | 413 | 36 | 4 | 9 | 231 | 39 |
| Rothia | 228 | 111 | 21 | 53 | 73 | 230 | 208 |
| Prevotella | 2 | 0 | 39 | 42 | 22 | 131 | 158 |
| Granulicatella | 12 | 16 | 6 | 34 | 215 | 18 | 14 |
| Luteococcus | 13 | 17 | 4 | 36 | 0 | 13 | 4 |
| Gemella | 7 | 4 | 6 | 1 | 15 | 4 | 19 |
|  |  |  |  |  |  |  |  |
|  | **Subject F** | | | | | | |
|  | **PT** | **10Gy** | **20Gy** | **30Gy** | **40Gy** | **50Gy** | **60Gy** |
| Streptococcus | 230 | 116 | 254 | 209 | 275 | 53 | 391 |
| Actinomyces | 38 | 127 | 418 | 84 | 71 | 60 | 480 |
| Veillonella | 81 | 172 | 29 | 38 | 1130 | 50 | 225 |
| Capnocytophaga | 800 | 577 | 146 | 424 | 119 | 1087 | 66 |
| Derxia | 58 | 64 | 5 | 8 | 40 | 77 | 194 |
| Neisseria | 697 | 1084 | 69 | 701 | 461 | 870 | 42 |
| Rothia | 22 | 249 | 286 | 365 | 616 | 293 | 227 |
| Prevotella | 13 | 21 | 7 | 45 | 0 | 12 | 173 |
| Granulicatella | 4 | 4 | 42 | 56 | 29 | 3 | 15 |
| Luteococcus | 58 | 46 | 65 | 6 | 15 | 6 | 5 |
| Gemella | 19 | 8 | 5 | 14 | 0 | 0 | 21 |
|  |  |  |  |  |  |  |  |
|  | **Subject G** | | | | | | |
|  | **PT** | **10Gy** | **20Gy** | **30Gy** | **40Gy** | **50Gy** | **60Gy** |
| Streptococcus | 422 | 141 | 173 | 200 | 134 | 95 | 612 |
| Actinomyces | 84 | 208 | 15 | 9 | 47 | 631 | 822 |
| Veillonella | 108 | 4 | 0 | 2 | 1 | 10 | 128 |
| Capnocytophaga | 408 | 353 | 51 | 242 | 232 | 26 | 39 |
| Derxia | 269 | 186 | 697 | 1459 | 989 | 510 | 7 |
| Neisseria | 439 | 259 | 565 | 145 | 138 | 90 | 0 |
| Rothia | 38 | 15 | 78 | 34 | 58 | 379 | 217 |
| Prevotella | 112 | 31 | 0 | 2 | 0 | 24 | 37 |
| Granulicatella | 183 | 13 | 23 | 17 | 7 | 0 | 4 |
| Luteococcus | 18 | 25 | 39 | 8 | 12 | 52 | 13 |
| Gemella | 9 | 21 | 0 | 2 | 5 | 2 | 65 |
|  |  |  |  |  |  |  |  |
|  | **Subject H** | | | | | | |
|  | **PT** | **10Gy** | **20Gy** | **30Gy** | **40Gy** | **50Gy** | **60Gy** |
| Streptococcus | 357 | 705 | 576 | 239 | 459 | 81 | 43 |
| Actinomyces | 205 | 286 | 131 | 102 | 421 | 1924 | 657 |
| Veillonella | 113 | 22 | 222 | 288 | 563 | 122 | 42 |
| Capnocytophaga | 55 | 78 | 267 | 242 | 64 | 89 | 174 |
| Derxia | 1193 | 1683 | 169 | 226 | 175 | 347 | 639 |
| Neisseria | 300 | 65 | 161 | 118 | 116 | 152 | 141 |
| Rothia | 1 | 0 | 315 | 174 | 284 | 20 | 9 |
| Prevotella | 2 | 0 | 61 | 100 | 80 | 2 | 0 |
| Granulicatella | 31 | 60 | 56 | 22 | 42 | 31 | 36 |
| Luteococcus | 2 | 0 | 30 | 10 | 4 | 7 | 1 |
| Gemella | 1 | 0 | 4 | 3 | 3 | 0 | 6 |
